# Characterization of the adiponectin promoter + Cre recombinase insertion in the Tg(Adipoq-cre)1Evdr mouse by targeted locus amplification and droplet digital PCR

**DOI:** 10.1080/21623945.2020.1861728

**Published:** 2020-12-29

**Authors:** Adrian M. Wong, Tushar P. Patel, Elizabeth K. Altman, Nicol Tugarinov, Giampaolo Trivellin, Jack A. Yanovski

**Affiliations:** a Section on Growth and Obesity and Division of Intramural Research, Eunice Kennedy Shriver National Institute of Child Health and Human Development (NICHD), National Institutes of Health, Bethesda, MD, USA; b Section on Genetics and Endocrinology, Division of Intramural Research, Eunice Kennedy Shriver National Institute of Child Health and Human Development (NICHD), National Institutes of Health, Bethesda, MD, USA; c Endocrinology Unit and Laboratory of Cellular and Molecular Endocrinology, Humanitas Clinical and Research Center – IRCCS, Rozzano (MI), Italy

**Keywords:** Adiponectin, adipose tissue, tissue-specific gene expression, insertion point, transgenic mouse, adipocyte

## Abstract

The Tg(Adipoq-cre)1Evdr mouse has become an important tool in adipose tissue biology. However, the exact genomic transgene integration site has not been established. Using Targeted Locus Amplification (TLA) we found the transgene had integrated on mouse chromosome 9 between exons 6 and 7 of *Tbx18*. We detected transgene-transgene fusion; therefore, we used droplet digital polymerase chain reaction to identify *Cre* copy number. In two separate experiments, we digested with BAMHI and with HindIII to separate potentially conjoined *Cre* sequences. We found one copy of intact *Cre* present in each experiment, indicating transgene-transgene fusion in other parts of the BAC that would not contribute to tissue-specific *Cre* expression. *Cre* copy number for Tg(Adipoq-cre)1Evdr mice can be potentially used to identify homozygous mice.

## Introduction

The Jackson Laboratory Tg(Adipoq-cre)1Evdr mouse, developed by Eguchi et al. [1], has become an integral tool in the field of adipose tissue biology, cited in over 200 publications to-date. This transgenic mouse expresses Cre recombinase under the control of the mouse adiponectin (*Adipoq*) promoter, allowing the expression of Cre exclusively in white and brown adipose tissues [1]. The transgenic mouse was created using a 245 kb RP23-90G21 bacterial artificial chromosome (BAC), that was modified so that *Cre* was placed under the control of the starting ATG of the A*dipoq* promoter [1]. Originally microinjected into pronuclei of fertilized one-cell stage FVB/NJ embryos, after multiple back-crosses onto C57BL/6J mice, a 2014 SNP panel analysis performed at the Jackson Laboratories [2] suggested that the transgene insertion site might be on chromosome 9, but the exact integration site was not established. As a result, the recommended assay to detect the presence of the transgene does not differentiate homozygous from heterozygous Tg(Adipoq-cre)1Evdr [2].

**Figure 1.**
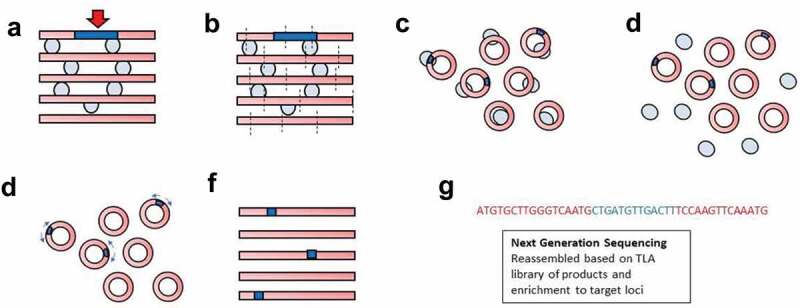
Schematic of TLA mapping approach. (a) Genomic DNA (red bars) containing a transgene (blue bar) with known sequence that has integrated at an unknown locus is crosslinked (light blue circles) preferentially to physically close genomic DNA and then (b) digested. (c) The resulting DNA fragments are then ligated to form small DNA circles containing different, nearby gDNA sequences. (d) The crosslinks are then reversed, separating the DNA circles. (e) ‘Inverse’ PCR is performed with primers (light blue arrows) internal to the transgene that face away from each other but amplify because of the circularization of the DNA. (f) The PCR products contain transgene fragments. Genetic loci in close proximity to the transgene are enriched due to fixation prior to digestion and ligation. (g) Fragments can then be sequenced by Next-Generation Sequencing and reassembled to identify integration sites and rearrangements

The large size of the transgene and limited information on its potential recombination site has made it difficult to determine the actual integration site using conventional sequencing methods. One approach to identify insertion sites is Targeted Locus Amplification in which DNA is selectively amplified and sequenced based on its crosslinking efficiency to sequences in close physical proximity (Figure 1) [3]. Genomic DNA is crosslinked to a gene of interest, digested, ligated, then reverse crosslinked to form small DNA circles that can be amplified by Inverse PCR. Sequences in close physical proximity to the gene of interest are highly enriched, and then the locus-specific sequences identified by next-generation sequencing can be reassembled and mapped against known chromosomal sequences. A single round of TLA mapping can potentially sequence genomic regions 70–100 kilobases in size [3].

Herein, we used TLA mapping to identify the insertion site of the transgene in the Tg(Adipoq-cre)1Evdr mouse. Due to the large size of the transgene, five rounds of TLA mapping – using five pairs of PCR primers internal to the transgene – were required to determine the first breakpoint of the transgene 5ʹ sequence. We found that the transgene was inserted between exons 6–7 of the *Tbx18* gene and that no genomic or transgene rearrangements occurred during recombination. Based on the number of reads of TLA mapping, we anticipated >1 copy of *Cre* might be present. We then used droplet digital polymerase chain reaction (ddPCR) to detect copy number variation of *Cre* and found only one functional copy of *Cre* present in hemizygous mice.

## Materials and methods

### Animal protocol

All animal experiments complied with the ARRIVE guidelines and were carried out in accordance with the National Institutes of Health guide for the care and use of laboratory animals, under Animal Study Protocol 17.053 approved by the NICHD Animal Care and Use Committee.

### Isolation of Splenocytes and TLA mapping

Male mice on chow diet that were hemizygous for A*dipoq-cre* (The Jackson Laboratory, Bar Harbour, ME B6.FVB-Tg(Adipoq-cre)1Evdr/J, Stock No: 028020) were sacrificed at 6–8 weeks of age and their spleens harvested. Spleen tissue was minced and passed through a 70 μM filter, then suspended in wash buffer made of 10% Foetal Bovine Serum (FBS) (Sigma St. Lewis, MO, catalogue #F2442) in phosphate-buffered saline (ThermoFischer Scientific, Waltham, MA, catalogue #70011069). Cells were spun for 5 minutes at 300G and the wash buffer was aspirated. 2 mL of red blood cell lysate buffer (ThermoFischer Scientific, Waltham, MA, catalogue #00-4333-57) was added and incubated at room temperature for 5 minutes. Cells were spun before the red blood cell lysate buffer was aspirated and the cells washed twice.

### TLA mapping

Splenocytes were frozen at 500,000 cells/mL density in cryopreservation media (70% FBS, 20% DMEM 10% RPMI) for TLA mapping (Figure 1) per Cergentis (Cergentis B.V., Utrecht, The Netherlands) company protocols [3,4]. Five primer pairs (Table 1) were used that spanned the BAC, with one primer pair internal to *Cre*. Fragments were aligned to mouse assembly GRCm38/mm10.

**Table 1.** Primer pairs spanning the BAC. Five primer pairs were used to amplify portions of the 245 kB of the RP23-90G21 BAC plus Cre recombinase, starting at the Adipoq promoter region (primer pair 1). FW: forward; RV: reverse

Primer pair	Binding position	Direction	Binding position	Sequence (5ʹ→3ʹ)
1	24 kb	RV	23520	AAGAGTTCTGGGTGTTGTTT
		FW	24117	TTTGTCTGCAATGCTTTACC
2	76 kb	RV	75922	TGGTTTGAGCAAGGTTATCA
		FW	76203	CTCTTGCTGTAAGGGAACAT
3	127 kb	RV	127236	GGAGTTGATTCGTCTCCTAC
		FW	127516	TGGAAACGTCTAGGCTTATG
4	Cre	RV	176000/480	ATTACGTATATCCTGGCAGC
		FW	176001/907	GGAGTTTCAATACCGGAGAT
5	225 kb	RV	223544	GAGAAGTGGAGACTAGCATG
		FW	223747	CTGTGACTTTGAGAACTTGC

### Determination of copy number

DNA was isolated from Splenocytes using a Qiagen DNeasy Blood & Tissue Kit for DNA extraction (Qiagen, Germantown, MD, catalogue #69504). To separate possible conjoined *Cre* sequences, the DNA was digested with DNA restriction enzymes with known sites in the targeting BAC sequence both 5ʹ and 3ʹ to the *Cre* sequence: HindIII (NEB, Ipswich, MA, catalogue #R0104S) in one experiment and BamHI (NEB, Ipswich, MA, catalogue R0136S) in a second experiment. Copy number was determined using ddPCR carried out according to Bio-Rad (Hercules, CA) specifications [5]. Taqman *Cre* Copy Number Assay probes (ThermoFisher, Waltham, MA, catalogue #4400291) were used to quantify intact *Cre*. The murine transferrin receptor protein 1 gene *Tfrc* (ThermoFisher, #4458367) was used as the diploid reference gene. The results were analysed using QuantaSoft (Bio-Rad) software.

### Tbx18 expression

Mice hemizygous for the *Adipoq-cre* construct and C57BL/6J mice on standard chow diet were sacrificed at 22 weeks of age, with epididymal white adipose tissue (eWAT) and hypothalamus collected. RNA was isolated using the Qiagen RNeasy Mini Kit (Qiagen, Germantown, MD, catalogue #74106) and RNA concentration was measured using a NanoDrop (Thermo Fisher) spectrophotometer. 1 μg of RNA was converted to cDNA using the iScript cDNA Synthesis Kit (Bio-RAD, Hercules, CA, catalogue #1708891). Tbx18 qPCR Primers (ORIGENE, Rockville, MD, catalogue #MP216696) and beta-actin primers (Integrated DNA Technologies #88318771 and #88318794) were used in a final concentration of 0.5 μM in 1x Sybr Green Master Mix (Thermo Fisher, Waltham, MA, catalogue #4309155) with 100 ng cDNA. RT-PCR was run in a Thermo Fisher Viia7 RT PCR system and the results were analysed using Quanta Studio 3 Software (Thermo Fisher).

## Results

### Verification of intact Cre

**Figure 2.**
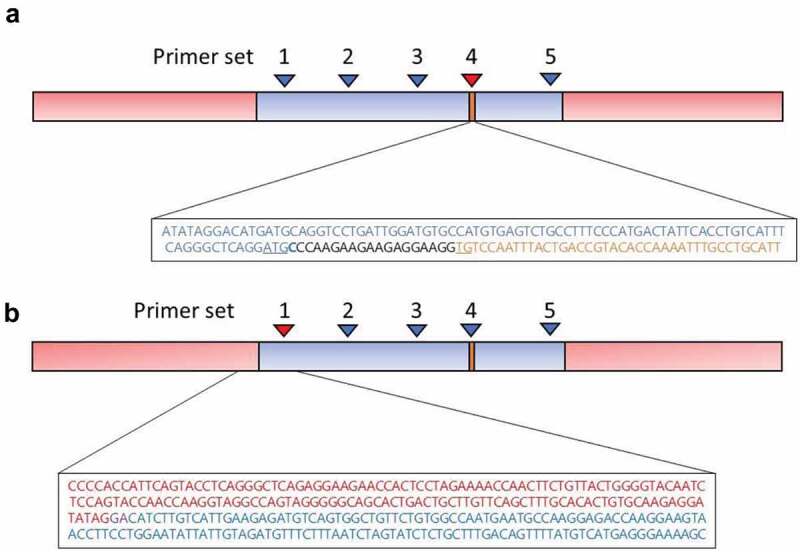
Results of TLA Mapping of the Tg(Adipoq-cre)1Evdr mouse BAC insertion point. (a) TLA mapping of the amplicons of primer set 4 (red arrowhead) internal to *Cre* (orange text) confirmed the inserted BAC sequence for *Adipoq* promoter (blue bar and in blue text) included a *Cre* sequence that was located after the starting ATG of the inserted BAC (underlined in blue text). After the BAC start codon, there was one Cytosine residue (bolded in blue text) belonging to the BAC, followed by 18 inserted bases (black), and then two TG residues (underlined in orange text) of the start codon of Cre recombinase (GenBank sequence MK854762), so *Cre* is transcribed in frame. (b) TLA mapping of the amplicons of primer set 1 (red arrowhead) identified the transgene (blue bar and blue text) integration site on chromosome 9 within the intron connecting exons 6 and 7of the *Tbx18* gene (red bar and red text), at a site with two homologous bases (purple text) found both in the normal genomic sequence and in the BAC. No recombination in the surrounding loci were detected

We first sought to verify that the *Cre* sequence was correctly inserted after the start site (ATG) of the adiponectin (*Adipoq*) gene, in-frame with the *Adipoq* promoter region. Using the alignment of sequences from Primer set 4 (the primer pair internal to *Cre*, Table 1), we found that at the position *Cre* was expected to integrate, *Cre* was fused to the *Adipoq* promoter with the *Cre* start codon (ATG) disrupted; however, there were additional inserted bases that made the sequence in-frame with the start codon of *Adipoq* (Figure 2(a)). As expected, the *Adipoq* promoter sequence was perfectly homologous with chr 16:23,155,228 to chr 16: 23,155,278 which corresponds to the native *Adipoq* gene, and places *Cre* immediately after the *Adipoq* promoter in the transgene.

### Identification of breakpoint sequences of the chromosomal insertion

Coverage across the transgene was high, with the highest density of coverage around the five primer pairs employed. The transgene integration site was identified on mouse chromosome 9 and the fusion reads were between chromosomal 9 locations 86,166,522 to 89,235,509. The break point of the transgene integration site was identified as being within the intron connecting exons 6 and 7 of *Tbx18*, where it had recombined at a site with two homologous bases shared by both the transgene and the reference chromosomal sequence (Figure 2(b)). No genomic rearrangements were detected in the region of the integration site. We also identified fusion sequences consisting of two parts of the transgene. A proprietary Cergentis script predicted that multiple copies of the transgene were present.

### ddPCR to determine Cre copy number

**Figure 3.**
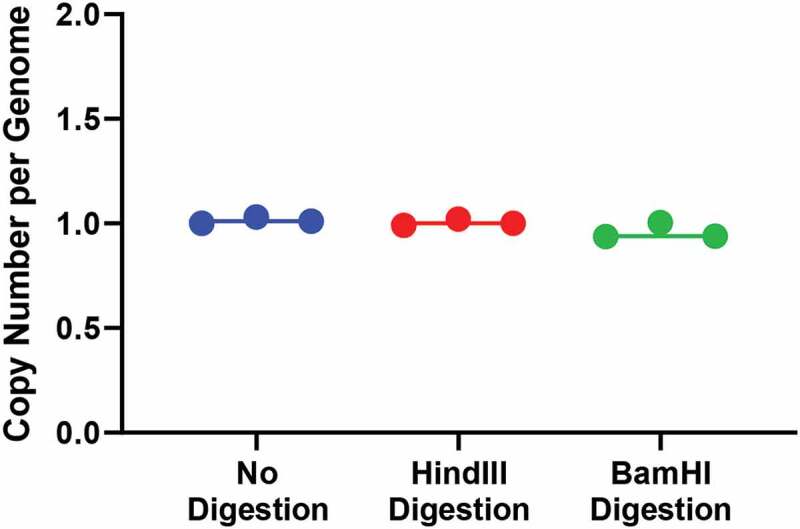
Results of ddPCR with undigested and digested genomic DNA. Results of ddPCR with undigested genomic DNA showed one copy per genome of *Cre* (blue circles and line). One copy per genome of *Cre* was detected by ddPCR after digestion with HindIII (red circles and line) and after digestion with BamHI (green circle and line). Results of three independent experiments for each digestion condition are shown

Because our TLA observations suggested that transgene-transgene fusion had occurred between different parts of the transgene, we wanted to determine the number of copies of *Cre* present per genome. We used primers internal to Cre and normalized the number of positive droplets detected to number of droplets found for the diploid *Tfrc* gene. Results suggested that one copy of intact *Cre* was present per genome (Figure 3). Because TLA mapping determined there was only one integration site, we reasoned that if there were multiple copies present with transgene-transgene fusion, they would be on the same allele and thus potentially not subject to proper ddPCR partitioning when intact gDNA was studied. We therefore digested the gDNA prior to ddPCR to separate potentially conjoined sequences. Using HindIII, which targets sites in the BAC sequence, only one copy of intact *Cre* was present per genome. Similarly, using BamHI, which cleaves at the 5ʹ end and 3ʹ end of *Cre* sites within the BAC, we still found only one copy per genome of intact *Cre* was present, making it likely that the transgene-transgene fusion detected by TLA mapping occurred in other parts of the transgene, and probably would not affect the ability of the transgene to express Cre recombinase. As a preliminary experiment, we also used ddPCR to determine *Cre* copy number of mice in five litters that were bred from a male and a female Tg(Adipoq-Cre)1Evdr hemizygous mouse (where both mice were found to have the Adipoq-Cre construct using conventional PCR). Surprisingly, we found no mice harbouring two copies of Cre (data not shown). It is unclear if it is possible to successfully breed homozygous mice, as Tbx18 is a key transcription factor in embryonic development. However, a larger sample size is required to determine this definitively.

## Conventional PCR across identified transgene insertion site

We attempted to design conventional PCR primers that would amplify the presence of the transgene by using the 3ʹ Tbx18 sequence identified as being immediately upstream of the insertion for the forward primer and the sequence identified as the most 5ʹ transgene insertion sequence for the reverse primer. We also created primer pairs across the entire Tbx18 sequence. However, despite using 47 primer sets (Table S), we were completely unsuccessful at generating a specific PCR product, as determined by sequencing. This may be due to the fact that the 5ʹ transgene insertion sequence has a long stretch of DNA that has high homology to mouse chromosome 16, with the CRE cassette beginning 71,872 bases into the insertion, making it difficult to design effective conventional PCR primers.

### RT-PCR to measure Tbx18 expression

**Figure 4.**
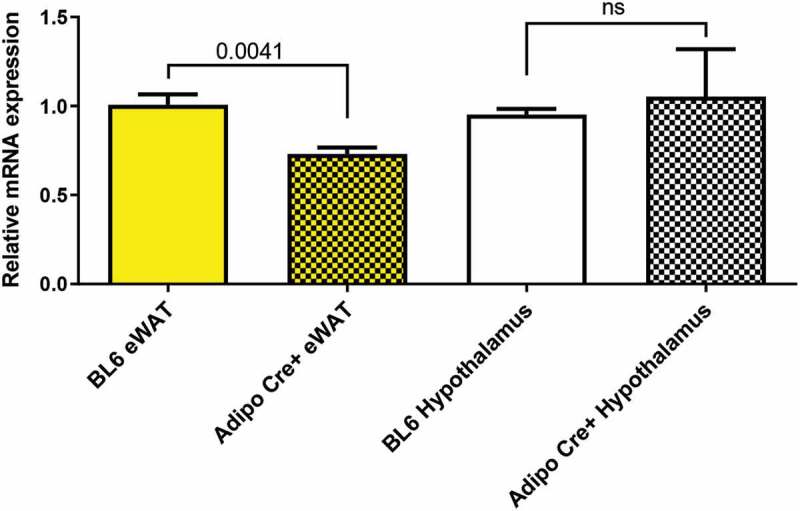
Tbx18 gene expression in BL/6 and Tg(Adipoq-cre)1Evdr mice. Results of quantitative RT-PCR expressed as the relative mRNA expression of *Tbx18* corrected for Beta-Actin. Epididymal white adipose tissue (eWAT) from mice hemizygous for *Adipoq-Cre* construct (yellow checkered bar) showed a 30% decrease in *Tbx18* expression from eWAT from BL/6 mice (yellow bar) (*p* = 0.0041). The hypothalamic expression in *Tbx18* in *Adipoq-Cre+* mice (black and white checkered bar) was not different from that of BL/6 mice (white bar)

Because we found the TG was inserted in the intronic region connecting exon 6 and 7, we wanted to determine if the large transgene insertion would lead to altered gene function, as large intronic insertions may alter gene expression. *Tbx18* is primarily expressed during embryonic development and is responsible for separation of anterior and posterior somite compartments [6]. However, according to the ProteinAtlas, *Tbx18* is expressed at low levels in white adipose tissue and brain in adult humans. We determined whether the insertion of the transgene would impair *Tbx18* expression (Figure 4). We found an approximately 30% decrease in *Tbx18* gene expression in eWAT collected from 22-week-old male mice hemizygous for *Adipoq-Cre*. We did not find a significant difference in hypothalamic *Tbx18* expression.

## Discussion

Understanding the genetics of the Tg(Adipoq-cre)1Evdr mouse is important for the field of adipose tissue biology because this mouse strain is used in numerous studies investigating the roles of adipose tissue proteins. The only other available adiponectin controlled *Cre* mouse available from Jackson Laboratory is a tamoxifen-inducible *Adipoq-Cre* that has not been as widely employed, with use only in 15 studies to-date. We identified the site of integration for the Tg(Adipoq-cre)1Evdr mouse transgene to be the intron between exons 6 and 7 of the *Tbx18* gene, an embryonic development gene that is important for the proper separation of anterior and posterior somite compartments [6] and specifically for proper myocardial function [7,8]. Importantly, we found no recombination in the surrounding region that would impact *Tbx18* function. TLA mapping also detected transgene-transgene fusion and we subsequently used ddPCR to identify the number of copies of intact *Cre* recombinase as one per transgene. Thus, mice that are hemizygous for the transgene produce CRE only from one copy of *Cre*. Hemizygous mice have been used in the vast majority of publications using the Tg(Adipoq-cre)1Evdr mouse because there has been no established technique for differentiating homozygous versus hemizygous mice. The ddPCR strategy may also prove useful for identifying copy number wherever classical PCR is not easily able to distinguish heterozygotes from homozygotes. The finding that only one copy of Cre is produced per genome in this mouse is important because it may explain the observation that when the hemizygous Tg(Adipoq-cre)1Evdr mouse is crossed with a mouse having a floxed gene there may be incomplete abolition or reconstitution in expression of the gene of interest [9,10]. The wide availability of reliable *Cre* TaqMan probes and the ability of ddPCR to determine *Cre* copy number easily means there is now a straightforward way to distinguish between hemi and homozygous Tg(Adipoq-cre)1Evdr mice. However, ddPCR reagents are costlier than those used for conventional PCR. For instance, as of November 2020, ddPCR SuperMix for 200 reactions is listed for 208 USD on the BioRad website, while the commonly used GoTaq SuperMix from Promega is listed for 349 USD for 1000 reactions. The website biocompare.com estimates the average cost of a ddPCR is 3 USD/reaction [11] while a 2015 Genetics and Molecular Biology paper projected the total cost of a conventional PCR reaction is 1.58 USD [12]. However, the cost of ddPCR can be reduced by using a conventional PCR assay to screen for the presence of the transgene and then screening for zygosity using ddPCR, thereby reducing the number of samples needed to run on ddPCR. Additionally, the two methods are comparable in total reaction time. In terms of accuracy, ddPCR is a more quantitative approach, providing a numerical copy number, while conventional PCR must be visually interpreted by the presence or absence of a band of interest. It is likely that homozygous mice would have greater CRE expression and would therefore be more likely to produce complete adipose-tissue-specific *Loxp* site recombination, but would likely also have a greater reduction in *Tbx18* expression. Further studies are needed to determine if this greater reduction in *Tbx18* expression would allow for successful embryonic development, given our difficulty in identifying homozygous Tg(Adipoq-cre)1Evdr mice.

## Supplementary Material

Supplemental Material
