# Supplementary material for: Characterization of the adiponectin promoter + Cre recombinase insertion in the Tg(Adipoq-cre)1Evdr mouse by targeted locus amplification and droplet digital PCR
Source: Adipocyte. 2020 Dec 29;10(1):21–7. doi: 10.1080/21623945.2020.1861728 (PMC7781622; doi:10.1080/21623945.2020.1861728)
Supplement: Supplemental Material [file KADI_A_1861728_SM9965.docx]

**Supplemental Table:**

| Forward Primer | | Reverse Primer | |
| --- | --- | --- | --- |
| Primer Sequence | Binding Location | Primer Sequence | Anticipated Binding Location |
| ACATTTCTCCCCCACCATTC | Tbx18 intron 6, expected to be upstream of the breakpoint | CCACTGACATCTCTTCAATGAC | Beginning of the insertion downstream of the breakpoint |
|  | Tbx18 intron 6, expected to be upstream of the breakpoint | ACAGCCACTGACATCTCTTC | Beginning of the insertion downstream of the breakpoint |
| CCCCACCATTCAGTACCTCA | Tbx18 intron 6, expected to be upstream of the breakpoint | TCCTTGGTCTCCTTGGCATT | Beginning of the insertion downstream of the breakpoint |
| CTTGTTCAGCTTTGCACACTG | Tbx18 intron 6, expected to be upstream of the breakpoint | TGTCTGCAATGCTTTACCGT | Beginning of the insertion downstream of the breakpoint |
| ACTTCTGTTACTGGGGTACAATC | Tbx18 intron 6, expected to be upstream of the breakpoint | TCCCACTTTTCCCTCATGACA | Beginning of the insertion downstream of the breakpoint |
| CAGAGGAAGAACCACTCCTAGA | Tbx18 intron 6, expected to be upstream of the breakpoint | GGTTACTTCCTTGGTCTCCTTG | Beginning of the insertion downstream of the breakpoint |
| GGTGCTTTGTTGCTCTCTCC | Tbx18 intron 6, expected to be upstream of the breakpoint | TCTGCAATGCTTTACCGTGA | Beginning of the insertion downstream of the breakpoint |
| TGTGAGTCCAAGGCTCTGTG | Tbx18 intron 6, expected to be upstream of the breakpoint |  |  |
| TGTGGTGCTTTGTTGCTCTC | Tbx18 intron 6, expected to be upstream of the breakpoint |  |  |
| CTCTGCGGACACAAATTAGGGAT | Tbx18 5' UTR |  |  |
| GAGGAAGCAGGAACGCG | Tbx18 5' UTR |  |  |
| GCTTCTTCGTCGGCCT | Tbx18 exon 1 |  |  |
| TGTTGCTGCTTCTCGGC | Tbx18 exon 1 |  |  |
| ATCGGAGTTGCCTTGAGG | Tbx18 intron 1 |  |  |
| CGGGGGCCCCTAAAA | Tbx18 intron 1 |  |  |
| GATGATCATCTCGGTGCCTATTTCG | Tbx18 exon 2 |  |  |
| TGCCAAGCCAGGTACG | Tbx18 exon 2 |  |  |
| GGGGGTTATAAAAAACCCACA | Tbx18 intron 2 |  |  |
| GTGTGTGTGTGTGGTGTCTACA | Tbx18 intron 2 |  |  |
| CTGTATCTCTTGTTGTCCACCGG | Tbx18 exon 3 |  |  |
| CGCATTGCTGGAAACATGCG | Tbx18 exon 3 |  |  |
| CGAGTTTGCACTCTGGGG | Tbx18 intron 3 |  |  |
| TTTGTATATGTTGCCTCCTGTGC | Tbx18 intron 3 |  |  |
| GGCTAAGCAAACCCTGAGTTTC | Tbx18 intron 3 |  |  |
| GGCCTTGGTCATCCAGC | Tbx18 exon 4 |  |  |
| CCACCATCCACTTAGAGCT | Tbx18 exon 4 |  |  |
| GGGAATTGCTCTTTTGTAGGTGACT | Tbx18 intron 4 |  |  |
| GTGGTGGACATTCAGGAAGG | Tbx18 intron 4 |  |  |
| GGCATATGCAATAGTGTCTGGGT | Tbx18 intron 4 |  |  |
| TTTCGCTTGGGCCCATCTAATA | Tbx18 intron 4 |  |  |
| CATTGGCTGTGCTTTAAAGGGG | Tbx18 intron 4 |  |  |
| TAGGCAGTGACAGTTGTGAAGA | Tbx18 exon 5 |  |  |
| GATGACATGCACACGTGGTT | Tbx18 exon 5 |  |  |
| GTTCCCTTCCACTCTAGGATG | Tbx18 intron 5 |  |  |
| GTAGCTCCACTTGGGAATTTG | Tbx18 intron 5 |  |  |
| GTTCCTCCCAGAGTCTCGG | Tbx18 exon 6 |  |  |
| GGATTCCTGTCTATCTTAAGGCG | Tbx18 exon 6 |  |  |
| CTCAGGGCTCAGAGGAAG | Tbx18 intron 6 |  |  |
| CAGGATAATTTGGGGAGTGGC | Tbx18 intron 6 |  |  |
| GCGGAATTGGGCTATTCTCAG | Tbx18 intron 6 |  |  |
| GCTTTGGGATTCCAGGGATA | Tbx18 exon 7 |  |  |
| GCCTCCAGAATGCGTATGACT | Tbx18 exon 7 |  |  |
| AGTGAGTTCCAGGACAGC | Tbx18 intron 7 |  |  |
| TGAGATGTCAGGAGGAGGTC | Tbx18 intron 7 |  |  |
| GATGTGTAGCAGGGACAGCAT | Tbx18 exon 8 |  |  |
| TGCCATACAATCCAACCCC | Tbx18 exon 8 |  |  |
| ATCCCTTCAGCTTCTCAGC | Tbx18 exon 8 |  |  |

**Supplemental Table: Primer pairs used unsuccessfully to generate a *Tbx18*+transgene product.** Conventional PCR primers were used to attempt to amplify the presence of the transgene by using the 3’ Tbx18 sequence identified as being immediately upstream of the insertion for the forward primer and the sequence identified as the most 5’ transgene insertion sequence for the reverse primer. We also created primer pairs across the entire Tbx18 sequence. However, despite using 47 primer pairs, we were completely unsuccessful at generating a specific PCR product as determined by sequencing of the PCR products.
